# Hexa-μ_2_-chlorido-μ_4_-oxido-tetra­kis­[(3-methyl-5-phenyl-1*H*-pyrazole-κ*N* ^2^)copper(II)]

**DOI:** 10.1107/S1600536810053663

**Published:** 2011-01-08

**Authors:** Hongshan He

**Affiliations:** a Center for Advanced Photovoltaics, Department of Electrical Engineering and Computer Science, South Dakota State University, Brookings, SD 57007, USA

## Abstract

The title compound, [Cu_4_Cl_6_O(C_10_H_10_N_2_)_4_], contains four Cu^II^ atoms which are bridged by six chloride anions. The central O atom is located on a crystallographic fourfold roto-inversion axis. Each Cu^II^ atom is coordinated by an N atom of a neutral monodentate 3-methyl-5-phenyl­pyrazole ligand, three Cl^−^ anions, and one O^2−^ anion. The geometry at each Cu^II^ atom is distorted trigonal–bipyramidal, with the three Cl^−^ ions in the equatorial plane and the N and O atoms in the axial positions.

## Related literature

For the formation of tris­pyrazolylborate anions, see: Tekeste & Vahrenkamp (2007 ▶); Jacobsen & Cohen (2004 ▶); Puerta & Cohen (2003 ▶); Parkin (2004 ▶). For the formation of dinuclear copper compounds, see: He & Sykes (2007 ▶). For the formation of tetranuclear compounds, see: Keij *et al.* (1991 ▶); Liu *et al.* (2003 ▶); Chiarella *et al.* (2009 ▶).
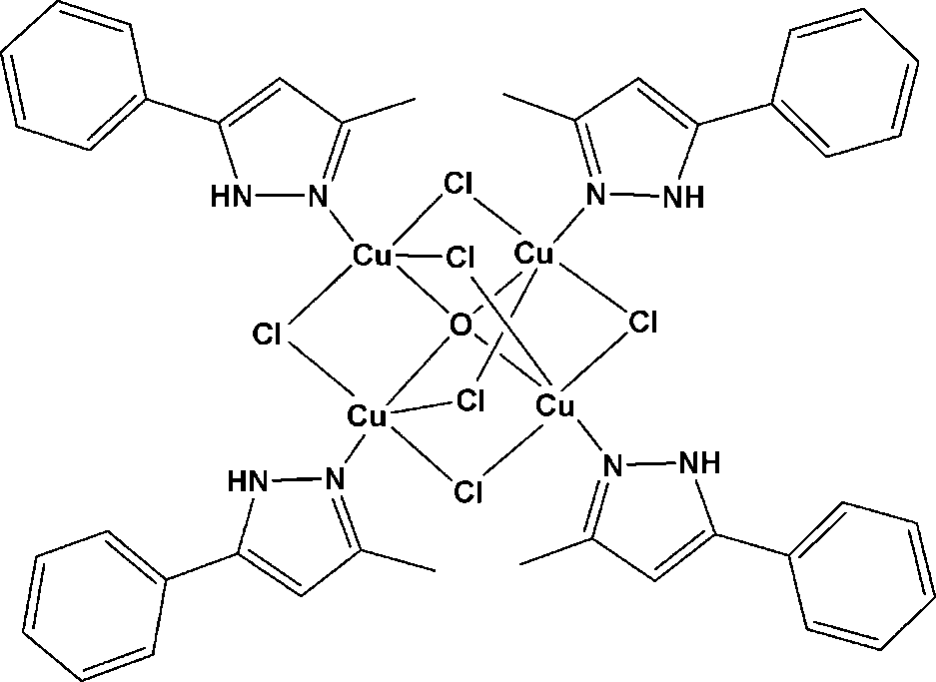


## Experimental

### 

#### Crystal data

[Cu_4_Cl_6_O(C_10_H_10_N_2_)_4_]
*M* *_r_* = 1115.66
Tetragonal, 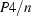

*a* = 14.5460 (6) Å
*c* = 11.1686 (7) Å
*V* = 2363.1 (3) Å^3^
*Z* = 2
Mo *K*α radiation
μ = 2.16 mm^−1^
*T* = 293 K
0.30 × 0.30 × 0.30 mm

#### Data collection

Bruker APEXII CCD area-detector diffractometer
Absorption correction: multi-scan (*SADABS*; Bruker, 2006 ▶) *T*_min_ = 0.564, *T*_max_ = 0.564
14047 measured reflections
2072 independent reflections
1184 reflections with *I* > 2σ(*I*)
*R*_int_ = 0.080

#### Refinement

*R*[*F* ^2^ > 2σ(*F* ^2^)] = 0.067
*wR*(*F* ^2^) = 0.194
*S* = 1.17
2072 reflections
136 parameters
H-atom parameters constrained
Δρ_max_ = 0.72 e Å^−3^
Δρ_min_ = −0.56 e Å^−3^

### 

Data collection: *APEX2* (Bruker, 2006 ▶); cell refinement: *SAINT* (Bruker, 2006 ▶); data reduction: *SAINT*; program(s) used to solve structure: *SHELXS97* (Sheldrick, 2008 ▶); program(s) used to refine structure: *SHELXL97* (Sheldrick, 2008 ▶); molecular graphics: *ORTEP-3 for Windows* (Farrugia, 1997 ▶); software used to prepare material for publication: *SHELXTL* (Sheldrick, 2008 ▶), *WinGX* (Farrugia, 1999 ▶) and *publCIF* (Westrip, 2010 ▶).

## Supplementary Material

Crystal structure: contains datablocks global, I. DOI: 10.1107/S1600536810053663/is2649sup1.cif

Structure factors: contains datablocks I. DOI: 10.1107/S1600536810053663/is2649Isup2.hkl

Additional supplementary materials:  crystallographic information; 3D view; checkCIF report

## Figures and Tables

**Fig. 1.**
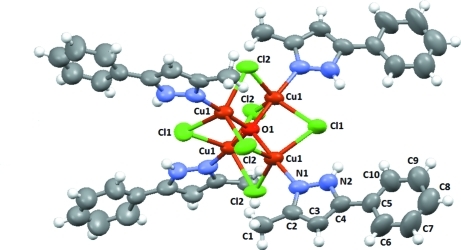
The structure of the title compound with displacement ellipsoids drawn at the 50% probability level. Hydrogen atoms are drawn as small circles of arbitrary radii.

## supplementary crystallographic information

### Comment

5-Methyl-3-phenylpyrazole has been widely used as starting material for the preparation of the trispyrazolylborate anion (Tekeste & Vahrenkamp, 2007; Jacobsen & Cohen, 2004; Puerta & Cohen, 2003; Parkin, 2004). It can form a dimeric complex (He & Sykes, 2007). Reported here is a new complex when it reacts with copper chloride.

In the title compound, (I), six chloride ions hold four copper ions together with an O atom encapsulated in the center (Fig. 1). The coordination geometry around each Cu^II^ is identical to each other with three Cl^-^ in the equatorial positions and N and O atoms in the axial positions. The distances between Cu1 and three Cl atoms are 2.361 (2), 2.514 (3) and 2.377 (2) Å. The distances between Cu1 and O1 and N1 are 1.9052 (10) and 1.953 (8) Å, respectively. The N1, Cu1 and O1 atoms fall almost in a line with an angle of 177.9 (5)°. The oxygen atom is located on a crystallographic fourfold roto-inversion axis.

### Experimental

5-Methyl-3-phenylpyrazole (16.0 mg, 0.1 mmol), prepared according to the literature (Puerta & Cohen, 2003), was dissolved in dichloromethane (10 ml) at room temperature. To this solution, copper(II) chloride dihydrate (8.7 mg, 0.05 mmol) in methanol (2 ml) was added. The resulting solution was stirred for two hours. The mixture was filtered and the filtrate kept at room temperature. Brown crystals were obtained after one week by slow evaporation.

### Refinement

All H atoms are geometrically constrained and refined in riding mode as follows: methyl *d*(C—H) = 0.96 Å, *U*_iso_(H) = 1.5*U*_eq_(C); aromatic *d*(C—H) = 0.93 Å, *U*_iso_(H) = 1.2*U*_eq_(C); *d*(N—H) = 0.86 Å, *U*_iso_(H) = 1.5*U*_eq_(N).

### Crystal data

**Table:**

[Cu_4_Cl_6_O(C_10_H_10_N_2_)_4_]	*D*_x_ = 1.568 Mg m^−^^3^
*M**_r_* = 1115.66	Mo *K*α radiation, λ = 0.71073 Å
Tetragonal, *P*4/*n*	Cell parameters from 16570 reflections
Hall symbol: -P 4a	θ = 2.3–25.0°
*a* = 14.5460 (6) Å	µ = 2.16 mm^−^^1^
*c* = 11.1686 (7) Å	*T* = 293 K
*V* = 2363.1 (3) Å^3^	Block, brown
*Z* = 2	0.30 × 0.30 × 0.30 mm
*F*(000) = 1124	

### Data collection

**Table:**

Bruker APEXII CCD area-detector diffractometer	2072 independent reflections
Radiation source: fine-focus sealed tube	1184 reflections with *I* > 2σ(*I*)
graphite	*R*_int_ = 0.080
φ and ω scans	θ_max_ = 25.0°, θ_min_ = 2.3°
Absorption correction: multi-scan (*SADABS*; Bruker, 2006)	*h* = −17→17
*T*_min_ = 0.564, *T*_max_ = 0.564	*k* = −17→17
14047 measured reflections	*l* = −13→13

### Refinement

**Table:**

Refinement on *F*^2^	Secondary atom site location: difference Fourier map
Least-squares matrix: full	Hydrogen site location: inferred from neighbouring sites
*R*[*F*^2^ > 2σ(*F*^2^)] = 0.067	H-atom parameters constrained
*wR*(*F*^2^) = 0.194	*w* = 1/[σ^2^(*F*_o_^2^) + (0.0526*P*)^2^ + 14.3478*P*] where *P* = (*F*_o_^2^ + 2*F*_c_^2^)/3
*S* = 1.17	(Δ/σ)_max_ < 0.001
2072 reflections	Δρ_max_ = 0.72 e Å^−^^3^
136 parameters	Δρ_min_ = −0.56 e Å^−^^3^
0 restraints	Extinction correction: *SHELXL97* (Sheldrick, 2008), Fc^*^=kFc[1+0.001xFc^2^λ^3^/sin(2θ)]^-1/4^
Primary atom site location: structure-invariant direct methods	Extinction coefficient: 0.0028 (8)

### Special details

**Table:**

Geometry. All e.s.d.'s (except the e.s.d. in the dihedral angle between two l.s. planes) are estimated using the full covariance matrix. The cell e.s.d.'s are taken into account individually in the estimation of e.s.d.'s in distances, angles and torsion angles; correlations between e.s.d.'s in cell parameters are only used when they are defined by crystal symmetry. An approximate (isotropic) treatment of cell e.s.d.'s is used for estimating e.s.d.'s involving l.s. planes.
Refinement. Refinement of *F*^2^ against ALL reflections. The weighted *R*-factor *wR* and goodness of fit *S* are based on *F*^2^, conventional *R*-factors *R* are based on *F*, with *F* set to zero for negative *F*^2^. The threshold expression of *F*^2^ > σ(*F*^2^) is used only for calculating *R*-factors(gt) *etc*. and is not relevant to the choice of reflections for refinement. *R*-factors based on *F*^2^ are statistically about twice as large as those based on *F*, and *R*- factors based on ALL data will be even larger.

### Fractional atomic coordinates and isotropic or equivalent isotropic displacement parameters (Å^2^)

**Table:**

	*x*	*y*	*z*	*U*_iso_*/*U*_eq_	
Cu1	0.72083 (7)	0.14541 (7)	0.90461 (11)	0.0567 (5)	
Cl2	0.57726 (15)	0.14864 (15)	1.0031 (3)	0.0729 (8)	
Cl1	0.7500	0.2500	0.7295 (3)	0.0705 (10)	
N2	0.6784 (5)	0.0507 (5)	0.6812 (8)	0.070 (2)	
H2	0.6890	0.1006	0.6424	0.084*	
N1	0.6896 (5)	0.0413 (5)	0.8019 (8)	0.063 (2)	
O1	0.7500	0.2500	1.0000	0.052 (3)	
C1	0.6666 (7)	−0.0801 (7)	0.9515 (11)	0.079 (3)	
H1A	0.6904	−0.0337	1.0042	0.119*	
H1B	0.7046	−0.1340	0.9560	0.119*	
H1C	0.6050	−0.0954	0.9749	0.119*	
C2	0.6663 (6)	−0.0449 (6)	0.8269 (10)	0.063 (3)	
C3	0.6412 (7)	−0.0918 (7)	0.7231 (11)	0.075 (3)	
H3	0.6232	−0.1530	0.7177	0.090*	
C4	0.6481 (7)	−0.0296 (7)	0.6292 (11)	0.072 (3)	
C5	0.6268 (7)	−0.0344 (7)	0.5035 (11)	0.075 (3)	
C6	0.5923 (10)	−0.1170 (9)	0.4580 (14)	0.120 (5)	
H6	0.5854	−0.1677	0.5079	0.143*	
C7	0.5686 (12)	−0.1228 (12)	0.3385 (15)	0.139 (6)	
H7	0.5476	−0.1786	0.3084	0.166*	
C8	0.5748 (10)	−0.0512 (12)	0.2651 (15)	0.122 (5)	
H8	0.5571	−0.0566	0.1854	0.146*	
C9	0.6072 (10)	0.0301 (11)	0.3078 (14)	0.120 (5)	
H9	0.6116	0.0811	0.2579	0.144*	
C10	0.6331 (9)	0.0355 (9)	0.4244 (13)	0.104 (4)	
H10	0.6568	0.0911	0.4516	0.125*	

### Atomic displacement parameters (Å^2^)

**Table:**

	*U*^11^	*U*^22^	*U*^33^	*U*^12^	*U*^13^	*U*^23^
Cu1	0.0436 (6)	0.0403 (6)	0.0863 (9)	−0.0028 (4)	−0.0010 (6)	−0.0064 (5)
Cl2	0.0424 (12)	0.0602 (14)	0.116 (2)	−0.0100 (10)	0.0079 (13)	−0.0219 (14)
Cl1	0.088 (2)	0.0405 (17)	0.082 (2)	−0.0115 (16)	0.000	0.000
N2	0.074 (5)	0.056 (5)	0.081 (6)	−0.014 (4)	0.002 (5)	−0.006 (4)
N1	0.051 (4)	0.043 (4)	0.096 (7)	−0.004 (3)	0.011 (4)	0.000 (4)
O1	0.039 (3)	0.039 (3)	0.077 (8)	0.000	0.000	0.000
C1	0.065 (6)	0.058 (6)	0.114 (10)	−0.016 (5)	0.001 (6)	0.009 (6)
C2	0.046 (5)	0.051 (5)	0.092 (8)	−0.001 (4)	0.009 (5)	0.008 (5)
C3	0.076 (7)	0.047 (5)	0.102 (9)	−0.013 (5)	0.000 (6)	−0.012 (6)
C4	0.066 (6)	0.054 (6)	0.097 (9)	−0.016 (5)	0.009 (6)	−0.012 (6)
C5	0.073 (7)	0.074 (7)	0.078 (8)	−0.017 (5)	0.013 (6)	−0.017 (6)
C6	0.149 (13)	0.090 (9)	0.119 (12)	−0.040 (9)	0.014 (10)	−0.039 (8)
C7	0.180 (16)	0.130 (14)	0.106 (13)	−0.057 (12)	0.008 (12)	−0.049 (11)
C8	0.108 (11)	0.142 (14)	0.115 (12)	−0.035 (10)	0.011 (9)	−0.029 (12)
C9	0.128 (12)	0.127 (12)	0.104 (11)	−0.037 (10)	−0.004 (9)	0.003 (10)
C10	0.122 (11)	0.096 (9)	0.095 (10)	−0.032 (8)	−0.014 (8)	0.004 (8)

### Geometric parameters (Å, °)

**Table:**

Cu1—O1	1.9052 (10)	C1—H1C	0.9600
Cu1—N1	1.953 (8)	C2—C3	1.394 (14)
Cu1—Cl2	2.361 (2)	C3—C4	1.388 (14)
Cu1—Cl2^i^	2.377 (2)	C3—H3	0.9300
Cu1—Cl1	2.514 (3)	C4—C5	1.439 (15)
Cl2—Cu1^ii^	2.377 (2)	C5—C10	1.350 (15)
Cl1—Cu1^iii^	2.514 (3)	C5—C6	1.397 (15)
N2—N1	1.365 (11)	C6—C7	1.38 (2)
N2—C4	1.377 (11)	C6—H6	0.9300
N2—H2	0.8600	C7—C8	1.33 (2)
N1—C2	1.328 (10)	C7—H7	0.9300
O1—Cu1^iii^	1.9052 (10)	C8—C9	1.359 (18)
O1—Cu1^i^	1.9052 (10)	C8—H8	0.9300
O1—Cu1^ii^	1.9052 (10)	C9—C10	1.358 (18)
C1—C2	1.482 (15)	C9—H9	0.9300
C1—H1A	0.9600	C10—H10	0.9300
C1—H1B	0.9600		
			
O1—Cu1—N1	177.9 (3)	H1B—C1—H1C	109.5
O1—Cu1—Cl2	85.45 (7)	N1—C2—C3	110.7 (10)
N1—Cu1—Cl2	94.8 (2)	N1—C2—C1	121.5 (10)
O1—Cu1—Cl2^i^	84.98 (7)	C3—C2—C1	127.8 (9)
N1—Cu1—Cl2^i^	96.7 (2)	C4—C3—C2	106.9 (8)
Cl2—Cu1—Cl2^i^	120.86 (5)	C4—C3—H3	126.6
O1—Cu1—Cl1	85.08 (7)	C2—C3—H3	126.6
N1—Cu1—Cl1	93.0 (3)	N2—C4—C3	104.9 (9)
Cl2—Cu1—Cl1	119.98 (8)	N2—C4—C5	121.4 (10)
Cl2^i^—Cu1—Cl1	117.06 (8)	C3—C4—C5	133.6 (9)
Cu1—Cl2—Cu1^ii^	81.32 (8)	C10—C5—C6	115.7 (12)
Cu1^iii^—Cl1—Cu1	77.85 (12)	C10—C5—C4	126.0 (10)
N1—N2—C4	111.7 (8)	C6—C5—C4	118.3 (12)
N1—N2—H2	124.1	C7—C6—C5	119.6 (15)
C4—N2—H2	124.1	C7—C6—H6	120.2
C2—N1—N2	105.8 (8)	C5—C6—H6	120.2
C2—N1—Cu1	131.9 (8)	C8—C7—C6	122.0 (15)
N2—N1—Cu1	122.0 (6)	C8—C7—H7	119.0
Cu1^iii^—O1—Cu1	112.00 (7)	C6—C7—H7	119.0
Cu1^iii^—O1—Cu1^i^	108.22 (3)	C7—C8—C9	119.3 (16)
Cu1—O1—Cu1^i^	108.22 (3)	C7—C8—H8	120.3
Cu1^iii^—O1—Cu1^ii^	108.22 (3)	C9—C8—H8	120.3
Cu1—O1—Cu1^ii^	108.22 (3)	C10—C9—C8	118.9 (15)
Cu1^i^—O1—Cu1^ii^	112.00 (7)	C10—C9—H9	120.6
C2—C1—H1A	109.5	C8—C9—H9	120.6
C2—C1—H1B	109.5	C5—C10—C9	124.4 (13)
H1A—C1—H1B	109.5	C5—C10—H10	117.8
C2—C1—H1C	109.5	C9—C10—H10	117.8
H1A—C1—H1C	109.5		
			
O1—Cu1—Cl2—Cu1^ii^	1.12 (6)	Cl1—Cu1—O1—Cu1^ii^	119.21 (2)
N1—Cu1—Cl2—Cu1^ii^	−176.7 (3)	N2—N1—C2—C3	0.8 (10)
Cl2^i^—Cu1—Cl2—Cu1^ii^	82.50 (10)	Cu1—N1—C2—C3	174.0 (6)
Cl1—Cu1—Cl2—Cu1^ii^	−80.53 (11)	N2—N1—C2—C1	−177.8 (8)
O1—Cu1—Cl1—Cu1^iii^	0.0	Cu1—N1—C2—C1	−4.6 (13)
N1—Cu1—Cl1—Cu1^iii^	179.1 (2)	N1—C2—C3—C4	−1.1 (11)
Cl2—Cu1—Cl1—Cu1^iii^	81.86 (9)	C1—C2—C3—C4	177.3 (9)
Cl2^i^—Cu1—Cl1—Cu1^iii^	−81.80 (9)	N1—N2—C4—C3	−0.6 (11)
C4—N2—N1—C2	−0.1 (10)	N1—N2—C4—C5	176.7 (9)
C4—N2—N1—Cu1	−174.1 (6)	C2—C3—C4—N2	1.0 (11)
Cl2—Cu1—N1—C2	−60.9 (8)	C2—C3—C4—C5	−175.8 (11)
Cl2^i^—Cu1—N1—C2	61.0 (8)	N2—C4—C5—C10	−0.3 (18)
Cl1—Cu1—N1—C2	178.7 (8)	C3—C4—C5—C10	176.0 (13)
Cl2—Cu1—N1—N2	111.4 (6)	N2—C4—C5—C6	−177.1 (11)
Cl2^i^—Cu1—N1—N2	−126.7 (6)	C3—C4—C5—C6	−0.7 (19)
Cl1—Cu1—N1—N2	−9.0 (6)	C10—C5—C6—C7	1(2)
Cl2—Cu1—O1—Cu1^iii^	−120.66 (8)	C4—C5—C6—C7	178.0 (13)
Cl2^i^—Cu1—O1—Cu1^iii^	117.77 (8)	C5—C6—C7—C8	−2(3)
Cl1—Cu1—O1—Cu1^iii^	0.0	C6—C7—C8—C9	1(3)
Cl2—Cu1—O1—Cu1^i^	120.13 (9)	C7—C8—C9—C10	1(2)
Cl2^i^—Cu1—O1—Cu1^i^	−1.44 (8)	C6—C5—C10—C9	1(2)
Cl1—Cu1—O1—Cu1^i^	−119.21 (2)	C4—C5—C10—C9	−175.8 (13)
Cl2—Cu1—O1—Cu1^ii^	−1.45 (8)	C8—C9—C10—C5	−2(2)
Cl2^i^—Cu1—O1—Cu1^ii^	−123.01 (9)		

Symmetry codes: (i) −*y*+1, *x*−1/2, −*z*+2; (ii) *y*+1/2, −*x*+1, −*z*+2; (iii) −*x*+3/2, −*y*+1/2, *z*.
